# Parasitism as a Long‐Lasting Interaction—First Evidence From Paleozoic Corals

**DOI:** 10.1002/ece3.71804

**Published:** 2025-07-13

**Authors:** Mikołaj K. Zapalski, Jan J. Król, Julien Denayer, Michał Zatoń

**Affiliations:** ^1^ Faculty of Geology University of Warsaw Warszawa Poland; ^2^ Institute of Geology Adam Mickiewicz University in Poznań Poznań Poland; ^3^ Evolution & Diversity Dynamics Lab, Geology Research Unit University of Liège Liège Belgium; ^4^ Institute of Earth Sciences University of Silesia in Katowice Sosnowiec Poland

## Abstract

The peak of reef development in the middle Paleozoic (Silurian‐Devonian) resulted in a dense network of interactions between corals and their symbionts. Due to their skeletonization, fossil corals and sponges preserved past interspecific relationships very effectively. Macrosymbionts of typical Paleozoic reef builders—corals and stromatoporoid sponges were traditionally interpreted as their commensals or parasites, despite their unclear systematic affinities. While the interpretations of parasitism were mostly based on alterations of the host's skeleton, one of the important features of parasitism, its long duration, remained unevidenced so far. Here we report on a Middle Devonian (approx. 395 Ma) alveolitid coral (Anthozoa: Tabulata), *Mariusilites* sp. (from Ardennes, Belgium), hosting numerous extracellular metazoan endosymbionts (*Torquaysalpinx* sp.) and displaying growth banding. The host (coral) growth banding allows an estimate of its growth rate as 3–4 mm per year, and as a result, the duration of the interaction appeared to be at least more than a year. The long duration of the interaction, together with the host's skeletal modification, suggests that these endosymbionts were parasites. This is the first case where the duration (longevity) of the parasitism can be determined in the hosting Paleozoic bioconstructing organisms.

## Graphical abstract

The peak of reef development in the middle Paleozoic resulted in a dense network of interactions between corals and their symbionts. The long duration (longevity) of one of the most common relationships, parasitism, has remained unevidenced so far. Here we report on a tabulate coral infested by parasites, where, based on growth bands, the duration of the parasitism can be determined as at least over a year.

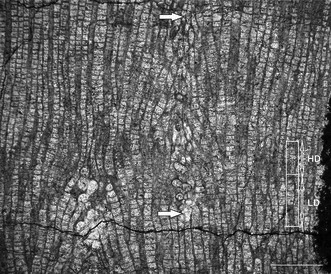

## 1 Introduction

Recent coral reefs are cradles of biodiversity and diverse interactions between organisms, and symbioses are at the core of all interactions (Vinn et al. 2025). Corals themselves host numerous metazoan symbionts, such as, for example, polychaetes, gastropods, and crustaceans (Barton et al. 2020; van der Schoot and Hoeksema 2024). The plethora of invertebrates associated with corals along a spectrum of symbioses ranges from mutualism to parasitism (Barton et al. 2020). The dynamics of these interactions can drive evolutionary change and influence evolutionary success or demise (Vinn et al. 2025). Without understanding the functioning of the coral holobiome (that includes a suite of micro‐ and macroorganisms), it is impossible to understand how corals react to a changing environment (Ainsworth et al. 2020). Also, understanding recent ecosystems may be incomplete without deep knowledge of their ancient counterparts.

Symbiotic relationships in the fossil record are best studied in those metazoans in which skeletal intergrowths allow observation of the *syn vivo* interactions of involved organisms (Tapanila 2008). Corals and sponges frequently hosted diverse symbionts, and due to their heavy skeletonization, preserve past interspecific relationships most effectively. Specifically, middle Paleozoic corals and stromatoporoid sponges hosted diversified multicellular organisms of unknown systematic position (Sokolov 1948; Oekentorp 1969). Such endobionts have been broadly distributed in all mid‐Paleozoic tropical seas since the Ordovician (Tapanila and Copper 2002), with the peak in the Middle Devonian (Tapanila 2005a, 2005b) and continuing until the latest Famennian (Zapalski et al. 2008; see recent review by Vinn et al. 2025). The systematic affinities of these organisms are poorly constrained, mostly because of their simple morphologies. As a result, they were usually classified either as *incertae sedis* structural fossils (e.g., Sokolov 1948) or ichnofossil taxa (Bertling et al. 2006). The most common include such (ichno) genera as *Chaetosalpinx, Torquaysalpinx, Helicosalpinx*, and *Phragmosalpinx* (Tapanila 2005a, 2005b).

The simple morphology of these organisms hampers their proper systematic assignment, yet the palaeoecological interactions have been intensively investigated for over 100 years (e.g., Clarke 1908; Oekentorp 1969; Plusquellec 1968). Older papers traditionally regarded interactions between these organisms and their hosts as commensal (e.g., Sokolov 1948; Oekentorp 1969), but more recent analyses showed that these relationships were closer to parasitism (Zapalski 2007, 2009). The parasitic nature of these endosymbionts was demonstrated based on the decrease of the host's growth rate, at least in the case of stromatoporoid hosts (Zapalski and Hubert 2011) or inferred perforation of the host's soft tissues (Zapalski 2007).

The incompleteness of the fossil record is one of the major problems in studies of past symbiotic relationships, and skeletal intergrowths provide an excellent opportunity to study such interactions. While various kinds of interactions can be detected in such intergrowths, one of the key features of parasitism, namely, long duration (Rózsa and Garay 2023) has not yet been demonstrated in coral‐symbiont relationships. In recent coral‐symbiont associations, the duration of the interaction is more explicit—for example, studies on a polychaete *Spirobranchus giganteus* living in *Porites* demonstrate that the symbiont lifespan may reach 40 years, as inferred from the host's growth bands (Nishi and Nishihira 1996).

Here we report on two specimens of Devonian tabulate corals (Anthozoa: Tabulata) infested by endosymbionts preserved together with the host's growth bands. As growth banding in modern corals is related to seasonality (Shinn 1966), these specimens allow us to calculate the host's growth rates and, as a consequence, temporal relationships between host corals and symbionts.

## 2 Materials and Methods

The present study focuses on two specimens of *Mariusilites* sp. (Tabulata, Favositida, Caliaporidae), one from Wancennes (inv. No. ULg.PA.WN II/57) and another from Wellin (inv. No. ULg.PA.WLM I/74) in Belgium. For the purpose of the present study, we have prepared seven thin sections, a standard method in fossil coral research, where a specimen is cut into thin (about 40 μm) translucent slices that allow observation of the internal structure (Ahmed and Vander Voort 2000). The thin sections were examined using a Keyence VHX‐7000 microscope at the Faculty of Geology, University of Warsaw, which allowed scanning the thin sections as well as taking measurements. The material is housed at the Animal and Human Paleontology Collections of the University of Liège, Belgium.

## 3 Geological Setting

During the Devonian Period, the Namur‐Dinant Basin, now part of the Ardennes Massif of southern Belgium, was situated on the southeastern margin of Laurussia in the Rheno‐Hercynian Ocean at tropical latitudes. The Namur‐Dinant Basin has a proximal facies in its northern part, whereas its southern part acted as a shallow basin with a more distal marine facies. In this area, the Eifelian (Middle Devonian) is characterized by mixed siliciclastic‐carbonate platform facies where several types of reefs developed. Their development is seemingly related to the synsedimentary interplay of tectonic blocks within the Namur‐Dinant Basin (Denayer 2019).

**FIGURE 1:**
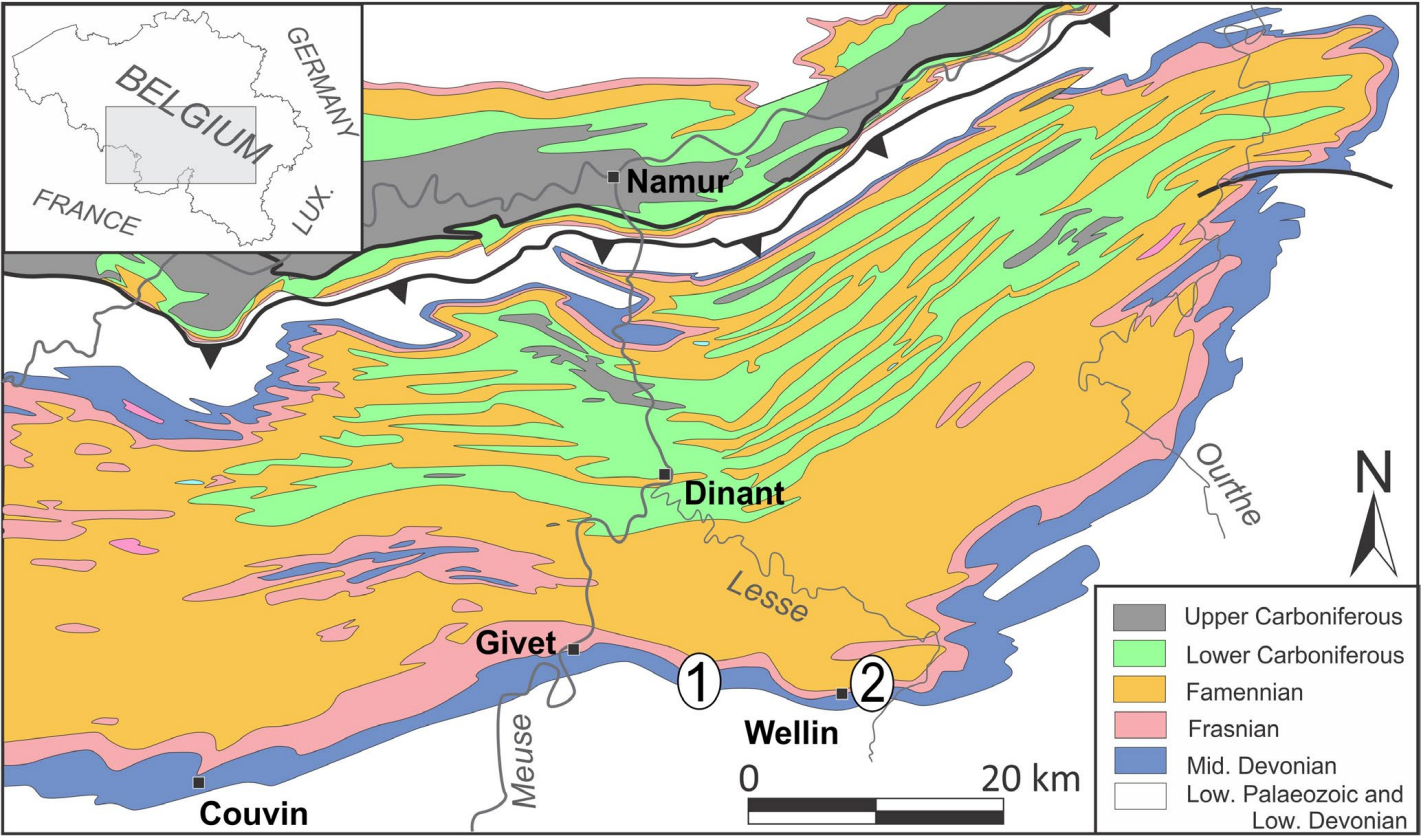
Simplified geological map with position of the two localities: 1—Wancennes, 2—Wellin “les Marlières.” Modified from Król et al. (2021).

The Wancennes locality (1 on Figure 1) exposes a large bioherm of Eifelian age. Though no diagnostic conodonts have been recovered from the bioherm, its age is constrained by underlying and overlying strata and is correlated with the *partitus* and *costatus* conodont zones (Denayer 2019, 2023). The bioherm starts with a light gray crinoidal rudstone covered by large lamellar stromatoporoids and becomes progressively richer in bulbous stromatoporoids and alveolitids upwards. The core facies of the bioherm is a light gray massive framestone with lamellar and bulbous stromatoporoids, ramose, lamellar, and dome‐shaped tabulate corals, and fasciculate and massive rugose corals. The matrix is micritic but rich in bioclasts, and cement‐filled cavities are common. The reef‐crest facies consists of large (up to 1 m in diameter) bulbous stromatoporoids associated with *Heliolites* and massive tabulate colonies. The present colony comes from this reef‐crest massive facies. The Wellin “les Marlières” locality (2 on Figure 1) exposes biohermal lenses interbedded with light gray crinoidal limestone known as the Wellin Formation and is late Eifelian in age (*ensensis* conodont Zone, Bultynck and Godefroid 1974). Its upper part is composed of a coarse‐grained limestone including two reefal beds or lenses, locally extremely rich in tabulate corals. One of these lenses is known as the “*Heliolites Field*,” already described by Król et al. (2021). The second specimen studied here comes from this locality.

## 4 Results

### 4.1 The Host

**FIGURE 2:**
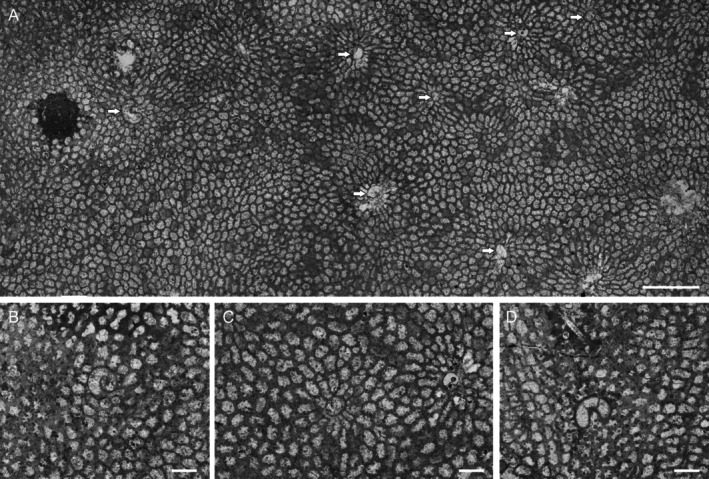
Transverse sections through *Mariusilites* sp. hosting *Torquaysalpinx* sp. Wancennes, Eifelian, specimen ULg.PA.WNII/57. (A) General view, arrows show symbionts; (B) detail, coiled *Torquaysalpinx* sp.; (C) detail, *Torquaysalpinx* sp., note the arrangement of the host corallites around the symbiont; (D) detail, coiled *Torquaysalpinx* sp. with diagnostic diaphragms visible. Scale bars—(A) 5 mm, (B–D) 0.5 mm.

**FIGURE 3:**
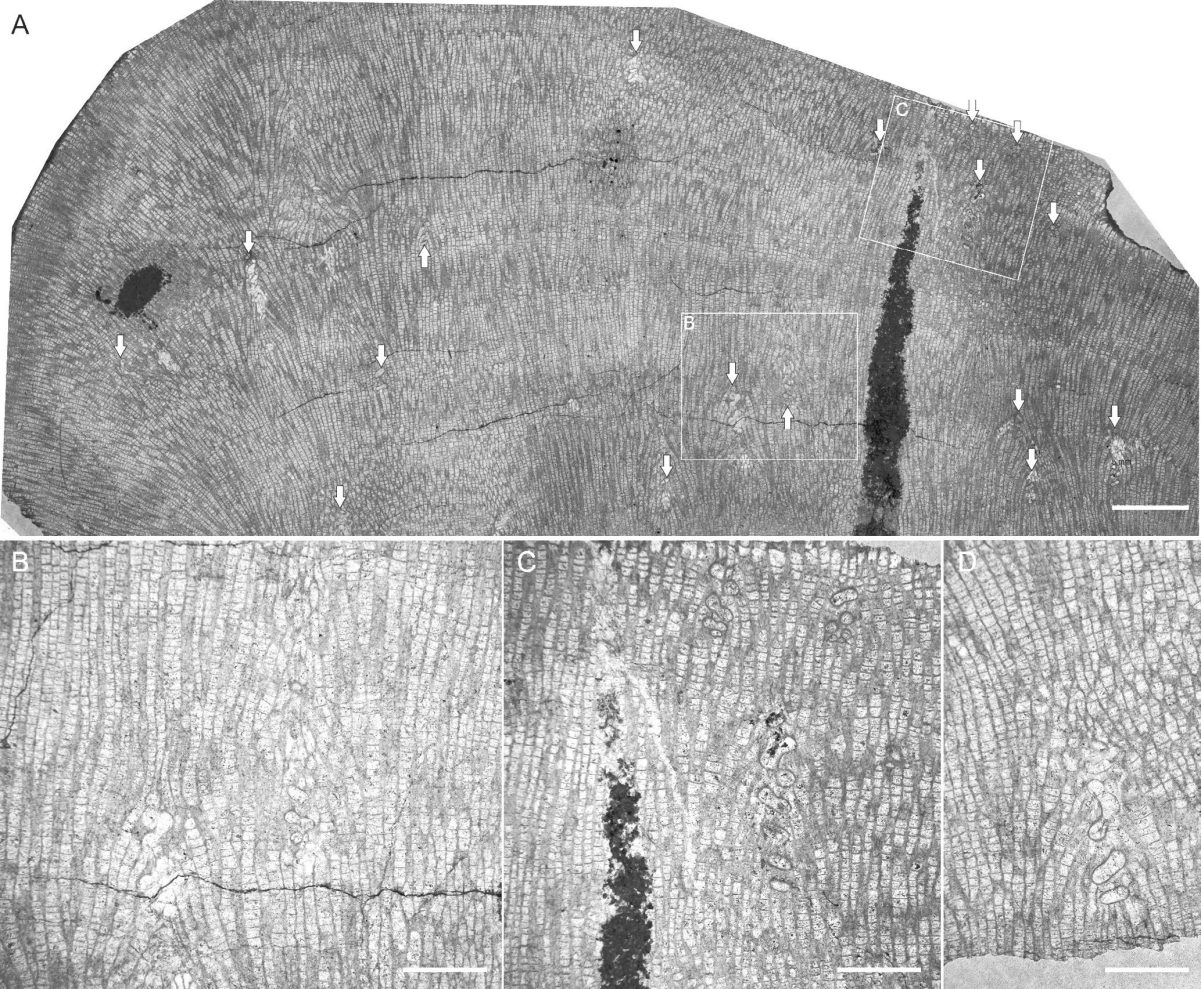
Longitudinal sections through *Mariusilites* sp. hosting *Torquaysalpinx* sp. Wancennes, Eifelian, specimen ULg.PA.WNII/57. (A) General view, arrows show endosymbionts; (B–D) detailed views. (B) Note the alteration of the host's corallites above the symbiont, see also Figure 5. Scale bars—(A) 5 mm, (B–D) 2 mm.

The corallum ULg.PA.WN II/57 from Wancennes is a small fragment measuring ~8.0 cm × 3.5 cm by 1.5 cm (Figures 2 and 3). Slender, prismatic corallites are oriented perpendicularly to the surface (Figure 2A). Squamulae are rare, short, and visible in some places on a transverse section; tabulae are complete, flat, or concave. These features allow the assignment of our specimen to the genus *Mariusilites* Mironova, 1974 (see Mironova 1974).

**FIGURE 4:**
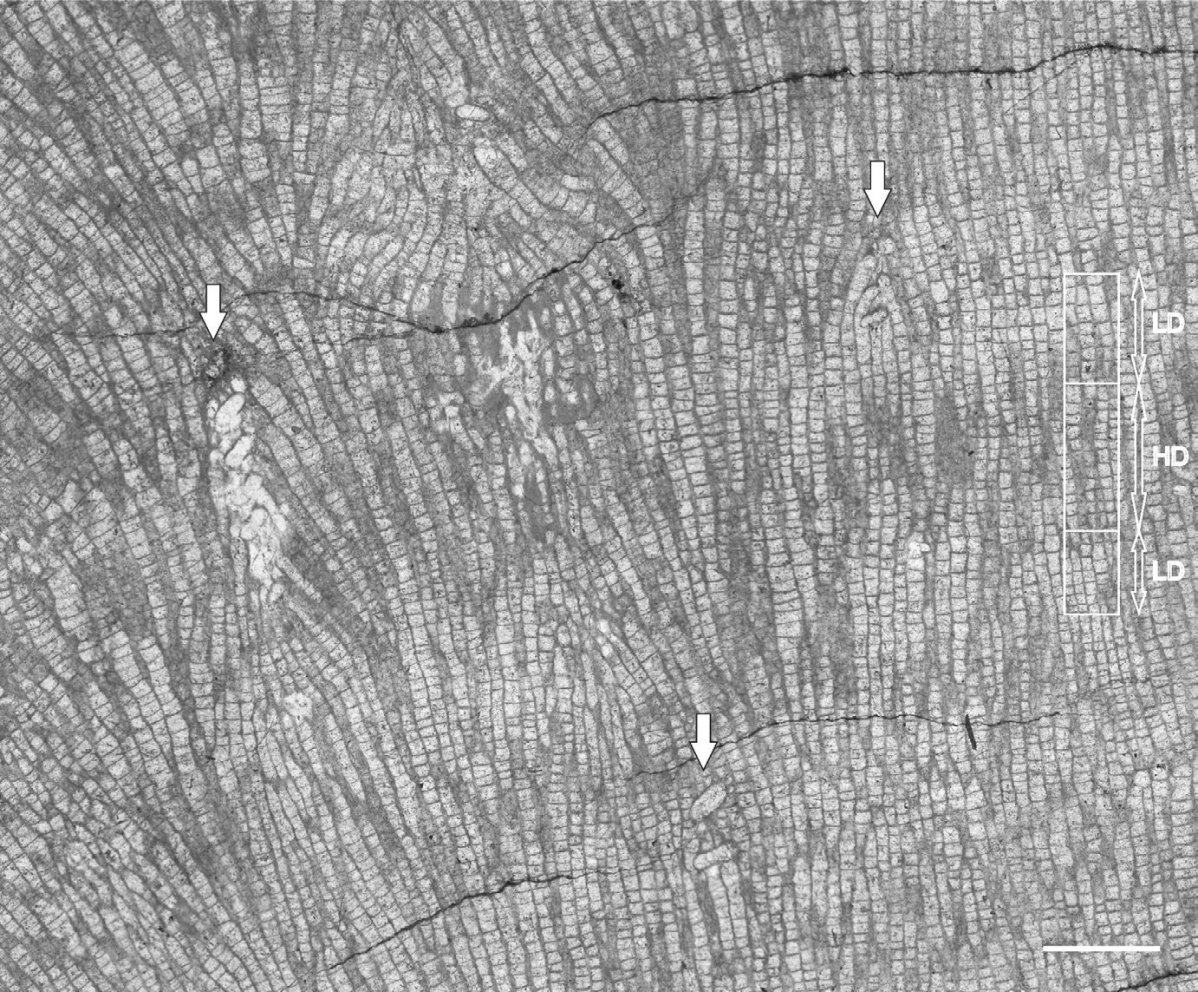
Longitudinal section through *Mariusilites* sp. Wancennes, Eifelian, specimen ULg.PA.WNII/57, arrows show *Torquaysalpinx* endosymbionts. In the right part of the figure, growth banding is marked, with examples of high (HD) and low (LD) density bands. The HD‐LD couplet reflects 1 year of growth. Scale bar: 2 mm.

**FIGURE 5:**
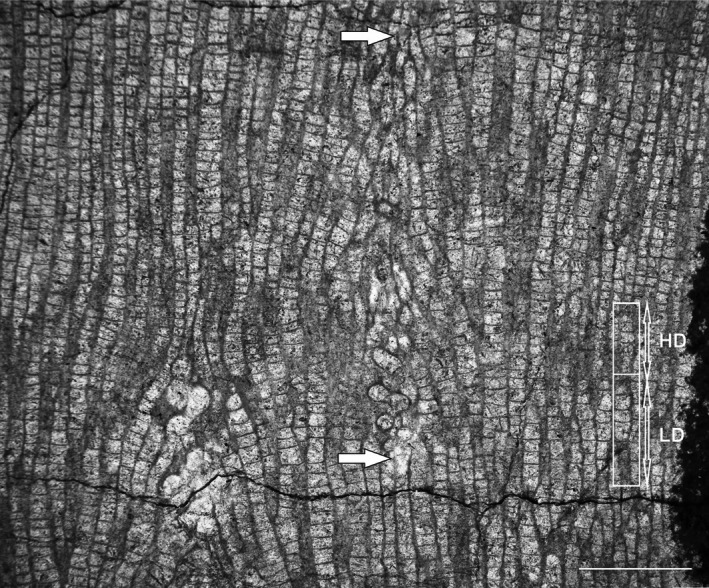
Longitudinal section through *Mariusilites* sp. Wancennes, Eifelian, specimen ULg.PA.WNII/57, host corallite alteration, same fragment as Figure 3B. The lower arrow marks the beginning of the *Torquaysalpinx* endosymbiont, the upper one marks where the host's corallite alteration ends. The endosymbiont tube likely deviates from the sectioning plane. In the right part of the figure, growth banding is marked, with examples of high (HD) and low (LD) density bands. The HD‐LD couplet reflects 1 year of growth. Scale bar: 2 mm.

The longitudinal section of the specimen reveals clearly visible dark (high‐density) and light (low‐density) growth bands. These dark and light growth bands are a result of differences in tabular spacing (Figure 3A, details on Figures 4 and 5). The entire growth band (dark and light band couplet) is usually about 3–4 mm thick, where the light band usually occupies 2/3 of the band thickness, but the transition between dark and light bands is gradual. The thickness of the dark (high‐density) band ranges from approximately 1.2 to 1.8 mm. The light band (low‐density band) thickness ranges from approximately 1.0 to 3.0 mm. The average tabular spacing in the dark band is 0.183 mm (±0.04 mm, *n* = 83), whereas in the light band it is 0.248 mm (±0.08 mm, *n* = 88). This colony is the main object of the present analysis, as it both displays growth banding and contains numerous endosymbionts.

Another colony, ULg.PA.WLM/74 from Wellin is a fragment (5.5 cm high) of a columnar colony, c. 3.9 cm in diameter. Its distal surface is not preserved. One growth band is visible (dark–light couplet). Because of the very fragmentary preservation of only three symbionts, this specimen was not analyzed further.

### 4.2 The Symbiont

The abundant endosymbionts have helicoidally coiled tubes that possess their lining (wall, Figure 2B,C). The diameter of the helix slightly increases from the bottom to the top. Their distribution seems to be random within the host colony, and in the longitudinal section, they appear at various growth stages of the host, as well as within various growth bands. In several sections, an internal diaphragm is also visible (Figure 2D). Helicoidal coiling is visible on longitudinal sections either as alternating tubes, if the section is closer to the helix axis, or as oval and toroidal shapes, if the section is closer to the helix perimeter (Figure 3B–D). These features allow us to assign our specimens to the genus *Torquaysalpinx* Plusquellec 1968. The external diameter of the tube is 0.5–1.4 mm, and the longest observed longitudinal section is about 4.5 mm. In most cases, the symbiont grew within one growth band, and very few reached the next growth band, at least as seen in the plane of thin section. They appear irregularly in different parts of the colony and also in different growth bands. Their measured length ranges from 1.2 to 4.7 mm (*n* = 11), usually 2–4 mm. The measured length is shorter than the tube itself, as the measurements are a result of the intersection between the section plane and fragments of the tube, whereas the endosymbiont tube may deviate from a single sectioning plane. The tubes alter the corallite arrangement immediately above the symbiont along the corallite growth axis (Figures 2A,B and 5), and the disturbance was visible on at least 7.7 mm of longitudinal distance (along the growth axes of corallites).

### 4.3 Host Modification

The presence of endosymbionts influenced the host's skeletal morphology. In the longitudinal section, corallites are bent towards the symbiont (Figure 3A–D), eventually covering the symbiont's tube, at least in the place of sectioning (Figure 5). This is visible in both longitudinal and cross‐sections, but not very clear in every cross‐sectioned symbiont. This is probably because the cross‐section was made through a somewhat weathered external part of the colony. Cross sections also reveal modification of the corallites in the immediate proximity of the symbiont. The corallites in cross‐section sometimes form regular chaplets, composed of corallites elongated in cross‐section, touching the symbiont on their narrower sides (Figure 2C). After the symbiont's growth cessation (its death or disappearance from the section plane), the neighboring corallites overgrew its remains (Figures 2B and 5). In the vicinity of the symbiont, the growth banding is altered.

## 5 Discussion

### 5.1 Growth Banding and the Age of the Host Colony

The growth banding is a distinct feature, visible in representatives of fossil and extant corals; it has been known since the papers by Ma (1933), and it is also well known in fossil tabulate corals (e.g., Scrutton and Powell 1980; Nowiński 1991; Young and Kershaw 2005; Zapalski et al. 2007), rugose corals (e.g., Scrutton 1964; Berkowski and Belka 2008) and scleractinians (e.g., Stanley Jr and Helmle 2010). It is generally accepted that couplets of dark–light zones represent annual increments (e.g., Wells 1963; Knutson et al. 1972; Weber et al. 1975; Lough and Cooper 2011). It can therefore be assumed that it is most probably also the case in tabulate corals. Therefore, the dark–light couplet can be interpreted as an annual increment. This implies that the annual extension rate was about 3–4 mm in the investigated specimen of *Mariusilites*. This is similar to other tabulate corals: typical heliolitids (2–6 mm/year) and less than typical favositids (5–18 mm/year; Scrutton 1998). Whereas the colony (WN II/57) is incomplete (the proximal part is eroded, but the distal surface is preserved in places), its lifespan can be estimated as at least 10–12 years.

The growth rates of modern scleractinians are similar to those of the *Mariusilites* investigated here. Dullo (2005) summarized typical growth rates of scleractinians and pointed out that it generally depends on the environmental setting, with depth being the most obvious factor. Among massive Caribbean forms, *Montastrea annularis* growth rates can, for example, range from 1.6 mm/year in the deepest settings (45 m deep) to about 12 mm in the shallows (0–3 m deep). Growth in various species of *Porites* can range from 2 to 36 mm/year, and *Siderastrea siderea* from 2.7 to 7.1 mm/year. In Indo‐Pacific species of the genus *Porites*, growth can range from 3 mm/year for the deep‐water (> 30 m) to 13 mm/year for the shallow‐water colonies (Dullo 2005).

### 5.2 The Growth and Distribution of the Endosymbionts

Comparison of the distribution of symbionts and the growth bands reveals that the symbionts usually span one dark–light couplet, or slightly more, and it can be inferred that they lived within the host corals for at least a year (Figures 4 and 5). They appear irregularly in different parts of the colony, also in different growth bands, and this suggests that there were numerous infestation events, and in most cases, the coral was able to overgrow the symbiont's tube. The capability of tabulate corals (particularly heliolitids) for successful overgrowth of their epibionts has been described by Król et al. (2018, 2021).

Modern corals often host numerous endosymbionts. Most of them, such as barnacles, bivalves, and polychaetes, do not grow above the surface of the hosts (van der Schoot and Hoeksema 2024). In the investigated case, the symbionts most probably also did not emerge above their host, and both the host and the symbiont were growing simultaneously. It seems unlikely that the symbionts settled on the colony surface, grew above the coral, and died within a short period of time, with their tubes being subsequently overgrown by the coral tissues over time. If the symbiont inside the calcareous tube died, it would be quickly bioeroded. In similar modern associations of vermetid gastropods–corals, the parasite may, in certain cases, emerge over the coral colony (e.g., Hoeksema et al. 2022), but the animal inside is alive, so the interaction is lasting.

### 5.3 Biological Affinities of the Endosymbionts

The characteristic helical coiling of *Torquaysalpinx*, the presence of an external wall and internal diaphragms are suggestive of some tentaculitoids—the small‐sized, lophophorate‐grade, tube‐dwelling animals (e.g., Vinn and Mutvei 2009; Taylor et al. 2010). Such tube characteristics are especially reminiscent of such tubeworms as trypanoporids and microconchids. Both had calcareous tubes. However, as only some of the microconchid species are known to have grown upward in a helical manner (Burchette and Riding 1977; Beus 1980; Vinn 2010a; Wilson et al. 2011; Zatoń and Mundy 2020; Opitek et al. 2024), trypanoporids seem to have invariably developed such coiling during their growth (Mistiaen and Poncet 1983; Weedon 1991; Tourneur et al. 1994). Additionally, whereas only some microconchid species possessed septa or diaphragms (Vinn 2006; Wilson et al. 2011), all trypanoporids known so far developed a variety of transverse and oblique peripheral septation (Weedon 1991). Thus, the organisms producing the *Torquaysalpinx* bioclaustrations would be more closely related to trypanoporids than to microconchids. Vinn (2016) even suggested that *Torquaysalpinx* may have descended from trypanoporids. Indeed, taking into account that both tubeworms are known exclusively from the Middle Devonian (e.g., Tapanila 2005a, 2005b; Vinn and Mutvei 2009), their phylogenetic relationship is even more probable. An extensive discussion on potential biological affinities of trypanoporids was provided by Tourneur et al. (1994).

The helical growth pattern in trypanoporids and microconchids most probably originated in response to such factors as higher sedimentation rate and/or overgrowth by other competing encrusters (e.g., Vinn 2010b). Trypanoporids are known from stromatolites (Mistiaen and Poncet 1983), so their helical upward growth let them keep pace with the growing microbialite they inhabited. So, residing within such upward‐growing organisms, as tabulates or stromatoporoids, would not have been a problem for such tubeworms. Once they colonized metazoans, they would have specialized and stayed obligatory symbionts. As in trypanoporids and some microconchids, the diaphragms present in *Torquaysalpinx* probably allowed maintenance of body size in a lengthening tube (Weedon 1991). However, the oblique peripheral septa, which probably played a wall‐strengthening function in trypanoporids (see Weedon 1991), have no longer been necessary for *Torquaysalpinx*, as the helically growing symbiont was encased within the host skeleton and thus naturally protected. As most probably filter‐feeding organisms, *Torquaysalpinx* symbionts could have relied on food particles propelled by the host's tentacles. It is worth mentioning that other tentaculitoids, cornulitids, lived symbiotically within stromatoporoid and tabulate hosts (Dixon 2010; Vinn and Mõtus 2012; Vinn 2016). Unlike *Torquaysalpinx*, however, cornulitids were facultative endobionts.

While the *Torquaysalpinx* endobionts are superficially similar to Recent vermetid gastropods by overall shape and coiling (in certain species, such as, for example, *Petaloconchus laurae*; Scuderi 2012; generally, vermetids have irregular shells), the distinct microstructure of vermetids allows us to rule out such an affinity.

### 5.4 The Nature of the Relationship

Tapanila (2008) stated that full intergrowth is the best indicator of *syn vivo* symbiosis. Tapanila (2008) regarded representatives of *Torquaysalpinx* as intermediate between intergrowth and embedment. However, due to the observed reaction of the host to the growing symbiont, their *syn vivo* relationship is unquestioned.

Commensal relationships in the fossil record have frequently been reported (e.g., de Gibert et al. 2006; Torres‐Martínez et al. 2021), but it has been demonstrated that commensalism is difficult to prove in the fossil record (Zapalski 2011). The interaction discussed here cannot be considered mutualistic, mostly because it caused structural changes of the host skeleton (e.g., Figure 2C,F,G). Such changes always cause energy expenditure for the host organism. Rózsa and Garay (2023) in their critical review emphasize that parasitism is a long‐lasting relationship. As demonstrated above, the interaction lasted in most cases for at least a year, and sometimes slightly longer, thus corroborating the described relationship as parasitism.

Modern vermetid gastropods may be a good functional analogue of such an interaction between a coral host and parasites. Vermetids may heavily infest their hosts; in some cases, the gastropod tube blocks the growth of polyps immediately underneath (Hoeksema et al. 2022), and they may cause stress and damage to the surrounding polyps, which may be enough to consider them as parasites (van der Schoot and Hoeksema 2024).

## 6 Conclusions

The annual growth rate of the Middle Devonian tabulate corals *Mariusilites* sp. from Ardennes (Belgium) is estimated as 3–4 mm/year. Numerous organisms, assigned here to the genus *Torquaysalpinx* of probable lophophorate‐grade tentaculitoid affinity, infest the host corals. Alteration of the host's anatomy allows us to interpret the symbionts as parasites of the coral. Analysis of parasite distribution allows estimation of the duration of the interaction—the parasites infest the host usually for about a year, or slightly longer—this is evidenced on the thin sections, but likely longer than that, thus supporting the relationship identification as parasitism, which is of long duration.

## Author Contributions

**Mikołaj K. Zapalski:** conceptualization (lead), investigation (equal), project administration (lead), supervision (lead), validation (equal), visualization (lead), writing – original draft (lead), writing – review and editing (equal). **Jan J. Król:** investigation (equal), resources (equal), writing – original draft (supporting), writing – review and editing (equal). **Julien Denayer:** resources (equal), visualization (supporting), writing – original draft (supporting), writing – review and editing (equal). **Michał Zatoń:** validation (equal), writing – original draft (supporting), writing – review and editing (equal).

## Conflicts of Interest

The authors declare no conflicts of interest.

## Data Availability

No specific data were produced for this research. Specimens are housed at the collections of the University of Liege, as stated in the manuscript.
